# Interfilament interaction between IMPDH and CTPS cytoophidia

**DOI:** 10.1111/febs.14624

**Published:** 2018-08-31

**Authors:** Chia‐Chun Chang, Gerson D. Keppeke, Li‐Ying Sung, Ji‐Long Liu

**Affiliations:** ^1^ Department of Physiology, Anatomy and Genetics University of Oxford UK; ^2^ Institute of Biotechnology National Taiwan University Taipei Taiwan; ^3^ Agricultural Biotechnology Research Center Academia Sinica Taipei Taiwan; ^4^ School of Life Science and Technology ShanghaiTech University China

**Keywords:** CTP synthase, cytoophidium, IMP dehydrogenase, live‐cell imaging, super‐resolution imaging

## Abstract

Inosine monophosphate dehydrogenase (IMPDH) and cytidine triphosphate synthase (CTPS) are two metabolic enzymes that perform rate‐limiting steps in the *de novo* synthesis of purine and pyrimidine nucleotides, respectively. It has been shown that IMPDH and CTPS can comprise a filamentous macrostructure termed the cytoophidium, which may play a role in regulation of their catalytic activity. Although these two proteins may colocalise in the same cytoophidium, how they associate with one another is still elusive. As reported herein, we established a model HeLa cell line coexpressing OFP‐tagged IMPDH2 and GFP‐tagged CTPS1 and recorded the assembly, disassembly and movement of the cytoophidium in live cells. Moreover, by using super‐resolution confocal imaging, we demonstrate how IMPDH‐ and CTPS‐based filaments are aligned or intertwined in the mixed cytoophidium. Collectively, our findings provide a panorama of cytoophidium dynamics and suggest that IMPDH and CTPS cytoophidia may coordinate by interfilament interaction.

**Abbreviations**
CTPS: cytidine triphosphate synthase
DAU: deazauridine
DON: 6‐diazo‐5‐oxo‐l‐norleucine
IMPDH: inosine monophosphate dehydrogenase
MPA: mycophenolic acid

## Introduction

The cytoophidium is a filamentous structure formed by metabolic enzymes. In past years, it has been shown that two key enzymes in the nucleotide *de novo* synthetic pathway, cytidine triphosphate synthase (CTPS) and inosine monophosphate dehydrogenase (IMPDH), are involved in cytoophidium formation in certain circumstances. For instance, treatment with IMPDH inhibitors, such as mycophenolic acid (MPA) and ribavirin, can trigger IMPDH filamentation, whilst conditions that impede glutamine‐dependent metabolism, such as glutamine‐deprived medium or treatment with glutamine analogues, induce both enzymes to form cytoophidia 1, 2, 3, 4, 5. Furthermore, IMPDH and CTPS cytoophidia were also observed in mouse and human tissues without drug induction, indicating filamentation of these enzymes is a natural physiological action *in vivo* 6, 7. Previous studies have also demonstrated that purified human IMPDH1 can form two types of octamer which are then able to combine to form a polymer structure *in vitro* 8. In addition, the formation of IMPDH cytoophidium has been shown to enhance GTP production in the cell 9. Similarly, human CTPS1 tetramer has recently been shown to be able to polymerise into strings to upregulate its enzymatic activity *in vitro* 10. Such protein polymers are considered to be the building blocks of the cytoophidium.

Assembly of the cytoophidium is an evolutionally conserved phenomenon. The CTPS cytoophidium was first reported in the fruit fly, and subsequently identified in bacteria, yeast and mammalian cells, whereas IMPDH cytoophidium has only been reported on mammalian models 1, 2, 11, 12, 13. In cultured cells, IMPDH cytoophidia were frequently observed in some cell types in normal conditions, while CTPS cytoophidia were rarely seen unless the cells were cultured in glutamine‐deficient medium or treated with certain drugs, such as glutamine analogues and deazauridine (DAU). To date, many more metabolic enzymes have been shown to be able to form similar filaments in various species 11, 14, 15. Although much about the regulation and function of the cytoophidium is still unclear, the formation of this structure has been widely accepted as a novel mechanism for fine‐tuning protein properties to adapt to intracellular and extracellular environmental changes 1, 7, 10, 16, 17, 18, 19.

In mammalian cells, linear cytoophidia can be as long as ~ 3–10 μm in length and ring‐shaped cytoophidia can be as big as ~ 2–5 μm in diameter 2, 3. The thickness of each cytoophidium can be up to more than 500 nm. Although IMPDH and CTPS can form cytoophidia independently, mixed IMPDH and CTPS cytoophidia are also frequently observed 7, 20. By electron microscopic analysis, the ultrastructure of the cytoophidium has been revealed as a bundle of fibres of protein polymer 13, 21. Yet, it is still elusive as to how these tiny fibres are organised in the macrostructure and how these two proteins coordinate.

In this study, we established a model HeLa cell line, which expresses both OFP‐IMPDH2 and CTPS1‐eGFP, for analysis of cytoophidium dynamics in real time. Based on this platform, we captured various movements of the cytoophidium, including assembly, disassembly, fusion and fission, providing important information for understanding its regulation, mobility and transportation. Moreover, we analysed the ultrastructure of IMPDH and CTPS mixed cytoophidia, revealing how the two enzymes form individual filaments and associate via interfilament interaction.

## Results

### Establishment of cell model for studying cytoophidium dynamics in live cells

**Figure 1:**
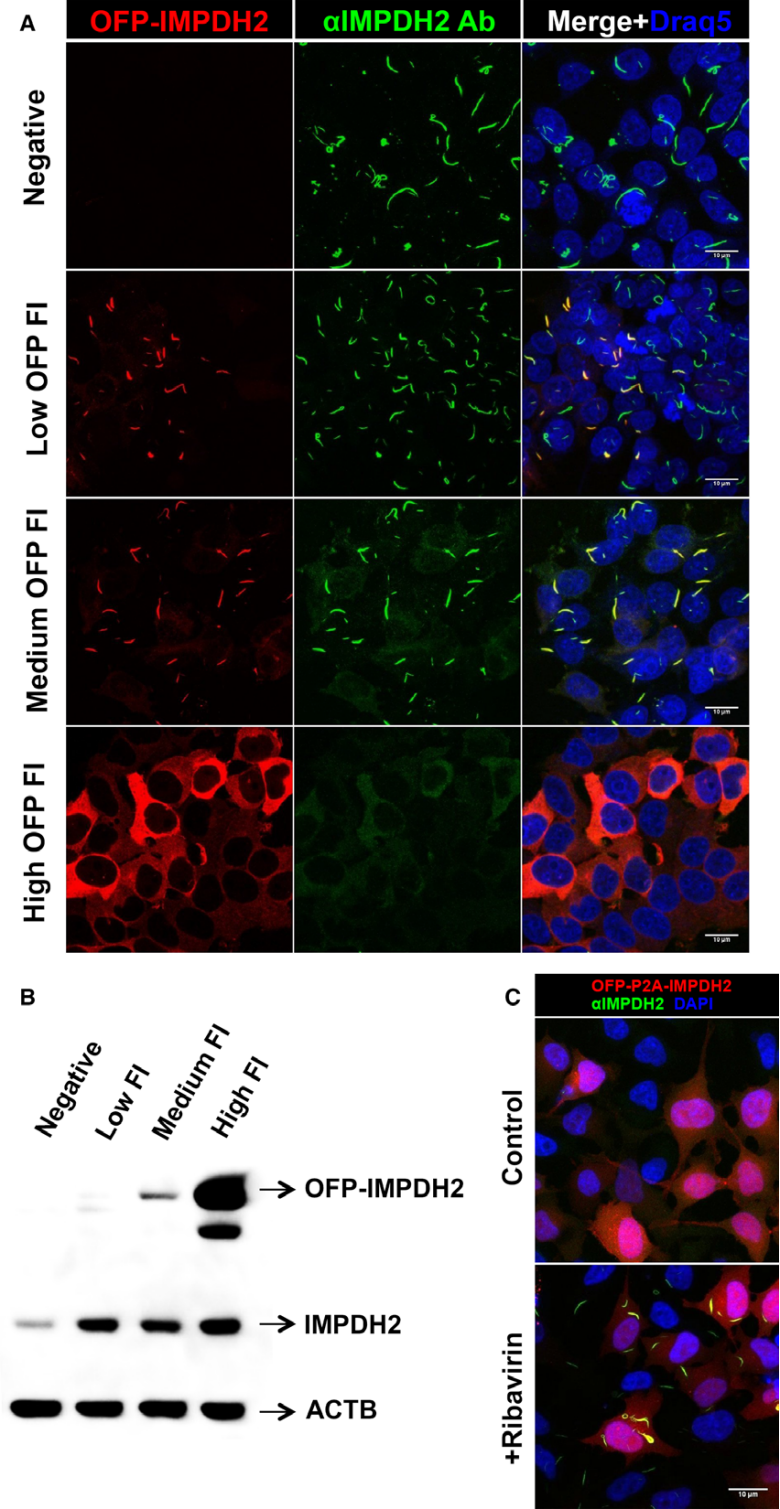
Cytoophidium assembly in cells overexpressing tagged IMPDH2. Hela cells transfected with OFP‐IMPDH2 construct and selected with Hygromycin B were sorted according to OFP fluorescence intensity (OFP FI). (A) Sorted cell groups were treated with 0.5 μm of ribavirin for 4 h before fixation and labelling with anti‐IMPDH2 antibody. (B) Sorted cell lysates were submitted to western blot for the analysis of IMPDH2 levels with and without OFP tag upon labelling with anti‐IMPDH2 antibody. (C) Immunofluorescence for IMPDH2 in OFP‐P2A‐IMPDH2‐expressing cells under conditions with and without ribavirin treatment showing that overexpression of nontagged IMPDH2 does not prevent cytoophidium assembly. Scale bars = 10 μm.

The cytoophidium is a dynamic structure since its size, shape and localisation change continuously. Immunostain‐based study can only acquire the morphology of the cytoophidium at the point of fixation. Visualising the cytoophidia in live cells could overcome such limitations and provide more information about how this large structure is organised and regulated. Therefore, we aimed to establish a cell model for capturing the localisation of both IMPDH and CTPS with confocal live‐cell imaging. To achieve this, we constructed the human IMPDH2 sequence with an OFPSpark tag at its N‐terminus and transfected HeLa cells with this plasmid. Consistent with a previous study 22, the cells expressing a high amount of OFP‐IMPDH2 were unable to form the IMPDH cytoophidium under IMPDH inhibitor treatment (Fig. 1A). However, in cells with a lower level of OFP‐IMPDH2, IMPDH normally assembled and disassembled according to the stimuli. Since drug‐induced filamentation is not affected by overexpression of nontagged IMPDH2, this could be caused by the fluorescent protein tag disturbing the protein interaction within the filament (Fig. 1A,C) 23. Thus, we chose HeLa cells stably expressing medium‐level fluorescence intensity of OFP‐IMPDH2 as the model cell line for live‐cell imaging (Fig. 1A,B).

### The cytoophidium is a flexible structure

**Figure 2:**
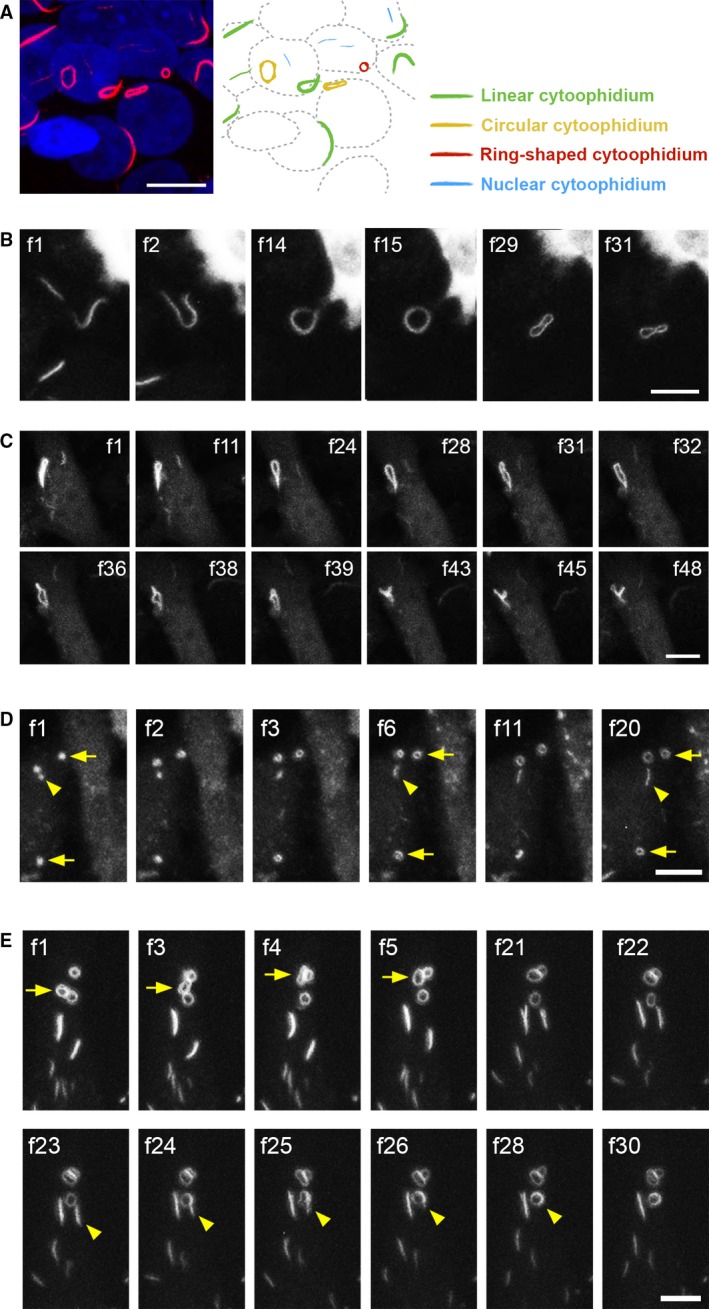
Transfiguration and fusions of IMPDH cytoophidia. (A) Normal HeLa cells were treated with 0.5 μm of ribavirin for 4 h before being fixed and labelled with anti‐IMPDH2 antibody (red) plus DAPI (blue). Linear, circular, ring‐shaped and nuclear cytoophidia are indicated by colour in the illustration. (B, C) OFP‐IMPDH2‐expressing HeLa cells were treated with DON (50 μg·mL^−1^) for 2 h before videos were recorded. A linear cytoophidium self‐fusing its two ends to become a circular cytoophidium is shown in (B). Representative frames from Video S1, showing a circular cytoophidium twisting into various shapes, are shown in (C). (D) OFP‐IMPDH2‐expressing HeLa cells were treated with MPA (100 μm) for 10 min to induce IMPDH cytoophidium assembly before recording Video S2. Growing ring‐shaped and linear cytoophidia are indicated by arrows and arrowheads, respectively. (E) Representative frames of Video S3 showing fission and fusion of MPA‐induced IMPDH cytoophidia. The fission and fusion of ring‐shaped cytoophidia are indicated by arrows and the fusion of linear and ring‐shaped cytoophidia is indicated by arrowheads. Each frame (f) was captured after a 2‐min interval for (B), (C), and (E), and a 1.5‐min interval for (D). Scale bars = 10 μm.

The cytoophidium is also called ‘rods and rings’, because cytoophidia in linear and circular shapes are frequently observed. Here, we roughly classified cytoophidia into four types: linear, circular, ring‐shaped and nuclear, according to their appearance or subcellular localisation (Fig. 2A). In order to understand how the formation of different kinds of cytoophidia is initiated, we recorded time‐lapse videos of OFP‐IMPDH2‐expressing HeLa cells upon treatment with the glutamine analogue, 6‐diazo‐5‐oxo‐l‐norleucine (DON), which is known as an effective inducer for both IMPDH and CTPS cytoophidia 3, 5, 20. According to previous studies on the development of IMPDH and CTPS cytoophidia in mammalian cells, the assembly of cytoophidia has been proposed with five phases: nucleation, elongation, fusion, bundling and circularisation 5, 24, 25.

At the beginning of our recording, we observed a massive number of dot‐like cytoophidia, which assembled, elongated and in some cases performed serial fusions, fitting the steps in this model and also consistent with the results of previous studies 5, 25. In addition, a linear cytoophidium can self‐fuse its two ends to transform into a circular cytoophidium (Fig. 2B). After this circularisation, some circular cytoophidia further twist into many kinds of secondary structure without breakage (Fig. 2C and Video S1). When it is forming the ring‐shaped cytoophidium, the circularisation phase might take place before the elongation phase, as we observed ring‐shaped cytoophidia growing from dot‐like initiating structures but not linear cytoophidia (Fig. 2D and Video S2). It has been shown that two linear cytoophidia can fuse side‐by‐side or end‐to‐end 5, 25. Interestingly, we found that ring‐shaped cytoophidia can also fuse with a ring‐shaped or even another linear cytoophidium (Fig. 2E and Video S3), suggesting cytoophidia in different shapes are substantially interchangeable.

### IMPDH and CTPS are associated via interfilament interaction in mixed cytoophidia

**Figure 3:**
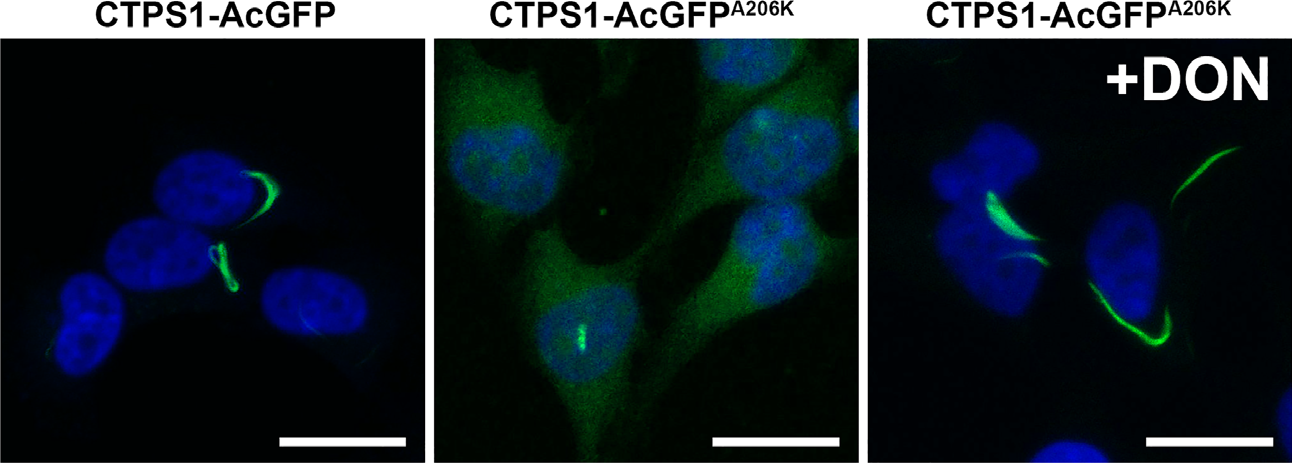
GFP dimerization promotes filamentation of CTPS1‐GFP fusion protein. The distribution of CTPS1‐GFP and CTPS1‐GFP^A^ ^206K^ proteins in transfected HeLa cells. Cytoophidia were frequently found in CTPS1‐GFP‐expressing cells, while the cytoophidium was rarely seen in CTPS1‐GFP^A^ ^206K^‐expressing cells under normal culture conditions. The CTPS1‐GFP^A^ ^206K^ fusion protein could be incorporated into the cytoophidium structure when the cells were treated with DON. Scale bars = 20 μm.

In order to assess the coordination between IMPDH and CTPS cytoophidia in live cells, we constructed a CTPS1‐GFP plasmid. After transfection, CTPS1‐GFP spontaneously assembled cytoophidia in some cells (Fig. 3). It has been reported that GFP‐derived fluorescent protein may dimerize at a physiological concentration, which may lead to abnormal protein aggregation 26. We therefore carried out site‐directed mutagenesis to generate a GFP A206K mutation so as to prevent GFP dimerization 27. Under normal conditions, CTPS1‐GFP^A206K^ formed far fewer cytoophidia than CTPS1 tagged with wild‐type GFP, and aggregated into the cytoophidium upon stimulation with DON (Fig. 3). This shows that expression of the CTPS1‐GFP^A206K^ fusion protein does not neither prevent nor promote CTPS cytoophidium assembly. Subsequently, we transfected the OFP‐IMPDH2 stable HeLa cell line with CTPS1‐GFP^A206K^ plasmid and performed live imaging to record the dynamics of both IMPDH and CTPS cytoophidia.

**Figure 4:**
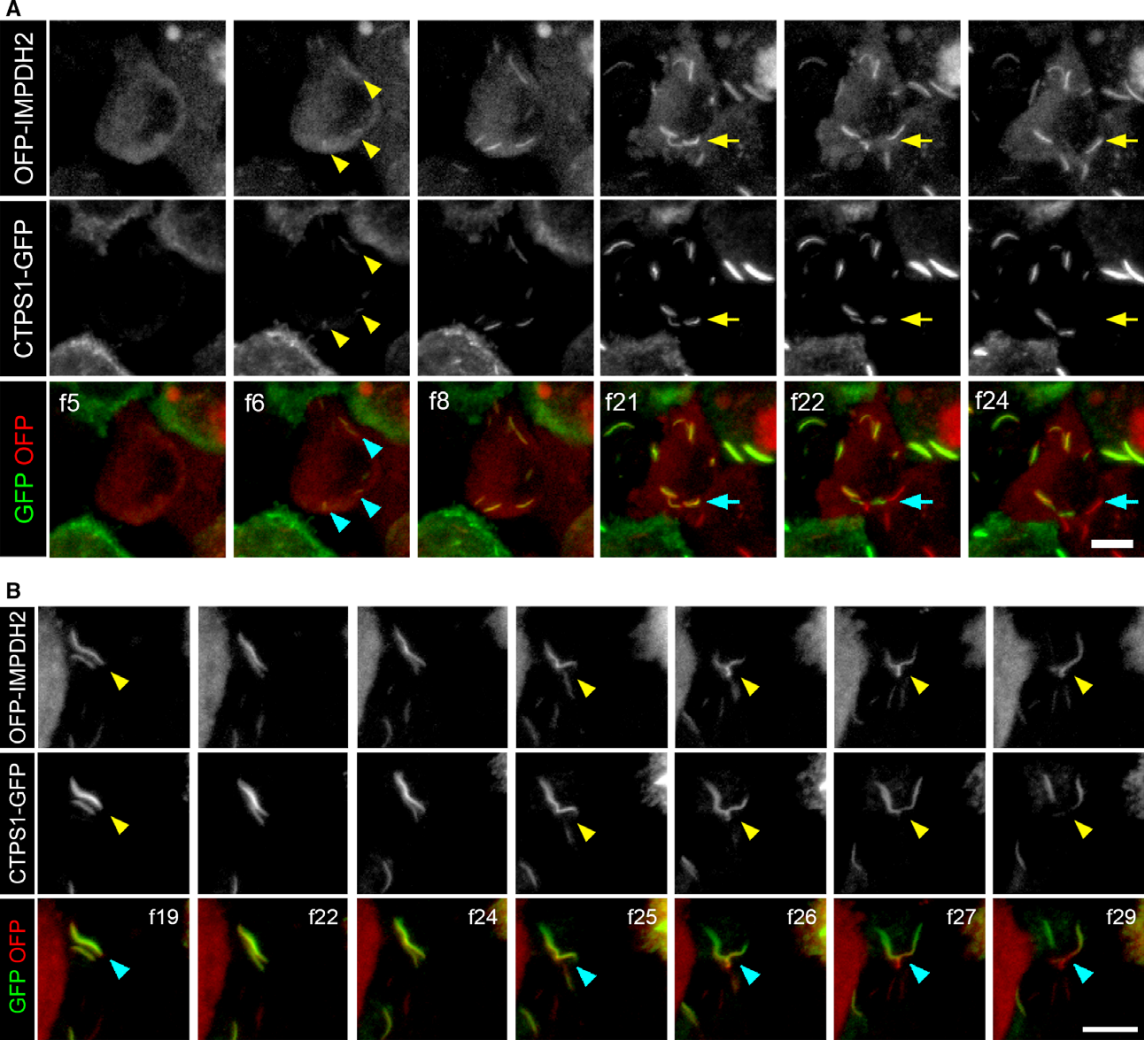
Formation and separation of IMPDH and CTPS filaments of a mixed cytoophidium. (A,B) Representative frames from Video S4 and S5, respectively. The cytoophidium formation in OFP‐IMPDH2 and CTPS1‐GFP coexpressing HeLa cells is shown. Videos S4 and S5 were recorded from 10 min after initiation of DON treatment (50 μg·mL^−1^) with a 2‐min interval between each frame. Cytoophidia in nucleation phase are indicated by arrowheads and the point of separation of CTPS and IMPDH cytoophidia is indicated by arrows in (A). The point of separation of CTPS and IMPDH cytoophidia is indicated by arrowheads in (B). Scale bars = 10 μm.

Upon DON treatment, the formation of IMPDH and CTPS was initiated within 20 min in some cells, and the colocalisation of the two proteins in cytoophidia was observed from the very beginning of the assembly (Fig. 4A f6–f21 and Video S4). As shown in the figure, the filaments of the two proteins have a similar shape and exhibited synchronous movement, suggesting they were associated in some aspect. However, in some cases, CTPS and IMPDH cytoophidia were suddenly separated from a mixed cytoophidium without a change in their appearance (Fig. 4, Videos S4 and S5).

**Figure 5:**
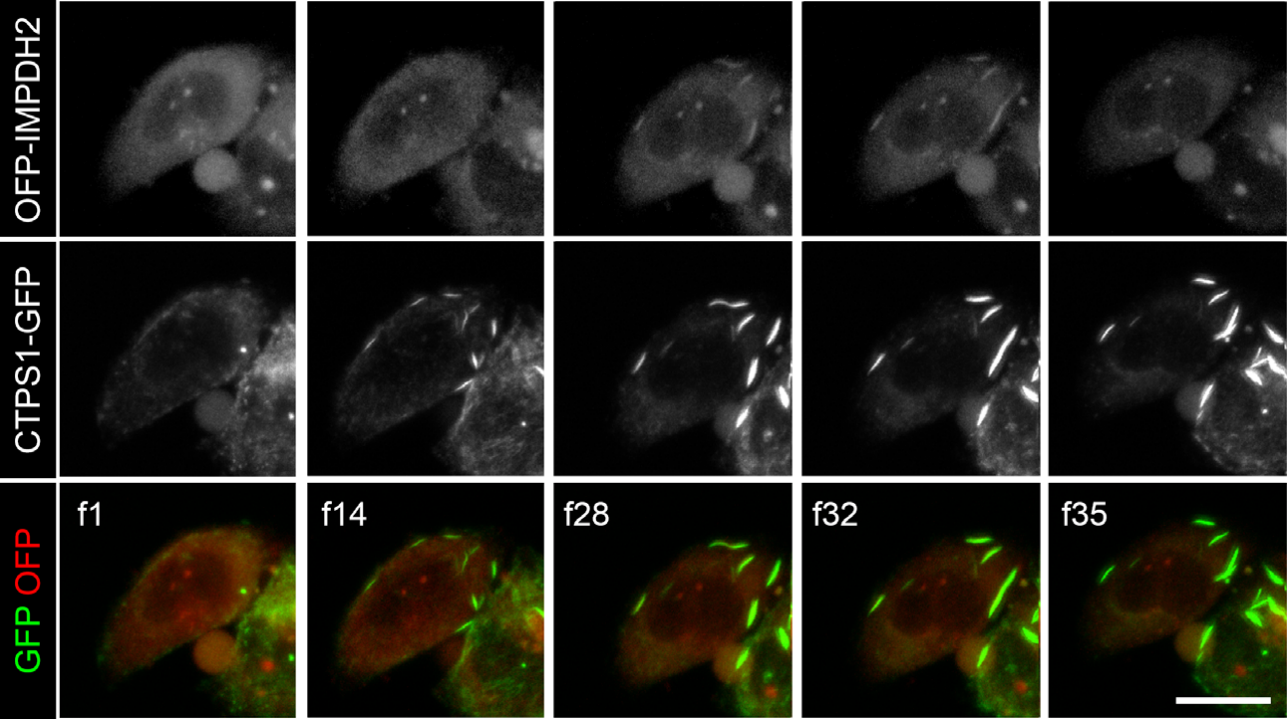
Assembly and disassembly of IMPDH filaments in the mixed cytoophidium. Representative frames of time‐lapse video of OFP‐IMPDH2 and CTPS1‐GFP‐overexpressing cells treated with DAU (100 μm) about 10 min before recording. Each frame was taken after a 2‐min interval. Scale bars = 20 μm.

We also examined cytoophidium formation after treatment with DAU, which is an inhibitor for CTPS and able to induce both CTPS and IMPDH cytoophidia formation within an hour of treatment in culture cells 7. However, as we previously reported, it also induces an elevation on intracellular GTP thereby promoting IMPDH cytoophidium disassociation after a longer period of time 7. Thus, with the DAU treatment, we were able to capture the formation of both filaments and also the disassembly of IMPDH cytoophidia. In the first 30 min of DAU treatment, CTPS formed long filaments alone (Fig. 5 f14). Then an intensive IMPDH signal showed up in existing CTPS cytoophidia for a short period before disappearing (Fig. 5 f28–f35). These findings suggest that even when they colocalise in the same cytoophidium, IMPDH and CTPS proteins were not mixed within filaments.

### Cytoophidium disassembly

**Figure 6:**
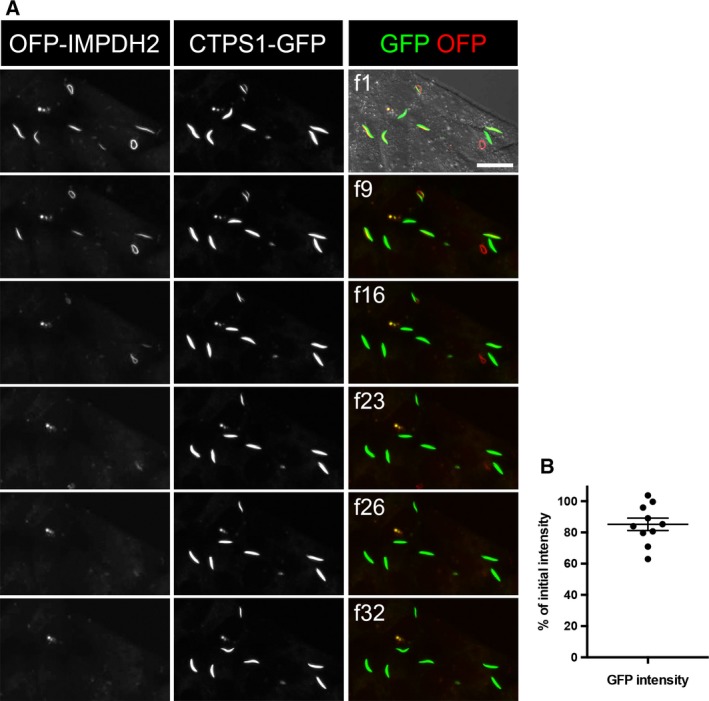
Disassembly of IMPDH in mixed cytoophidia does not dissociate CTPS filaments. (A) Representative frames of a video with frame numbers as indicated. After treatment with guanosine (100 μm), OFP‐labelled IMPDH filaments gradually disappear from DON‐induced mixed cytoophidia. Video was recorded from 30 min after initiation of guanosine treatment with a 2‐min interval between each frame. Scale bar = 20 μm. (B) Relative GFP intensity of each cytoophidium shown in frame 23.

**Figure 7:**
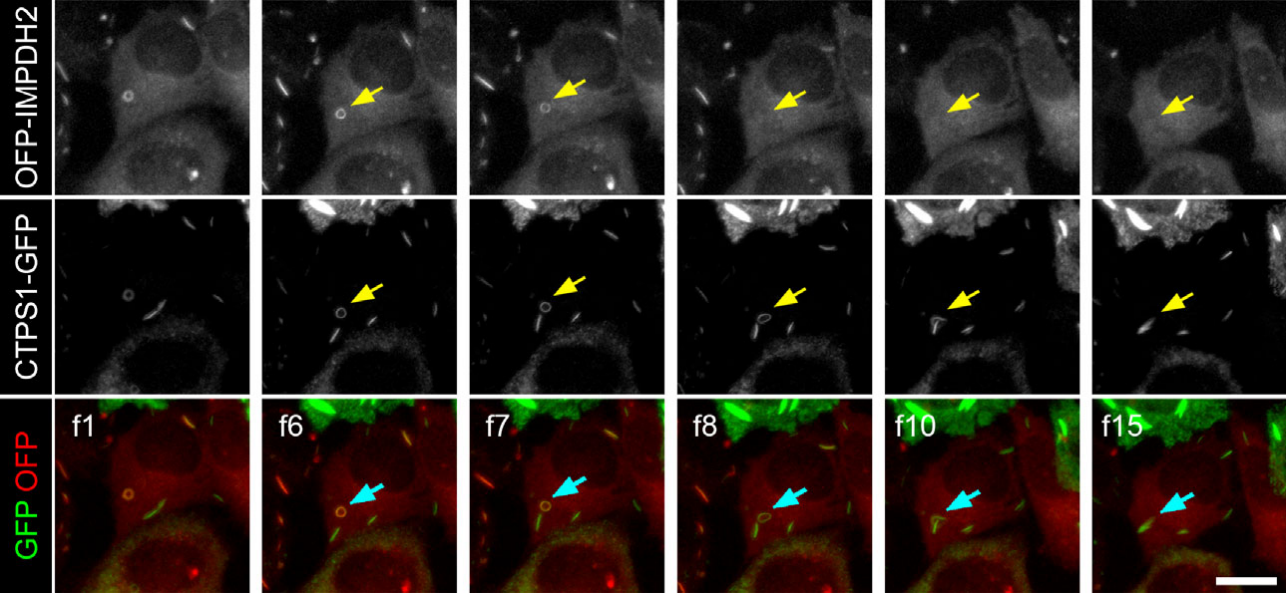
Disassembly of IMPDH filaments in the mixed cytoophidium triggers transfiguration of the cytoophidium. Representative frames of Video S6 show the morphological change of DAU‐induced an IMPDH/CTPS mixed cytoophidium after disassembly of IMPDH cytoophidium. Each frame was taken at a 2‐min interval. Scale bars = 20 μm.

**Figure 8:**
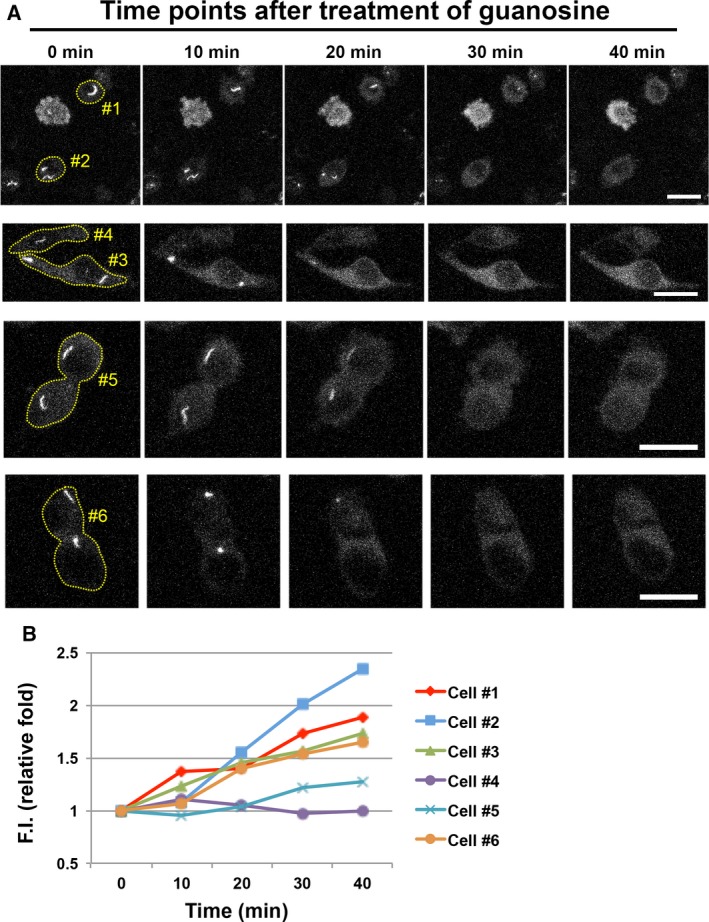
Quantification of fluorescence intensity of OFP‐IMPDH2‐expressing cells during cytoophidium disassembly. OFP‐IMPDH2‐expressing HeLa cells were treated with MPA (100 μm) for 6 h before live‐cell imaging to induce large IMPDH cytoophidia. Images were captured every 10 min upon supplementation of guanosine (1 mm) with the same setting of confocal microscope. Time‐laps images of each cell samples for analysis were shown in (A). (B) Quantification of fluorescence intensity (F.I.) of corresponding samples shown in (A). F.I. of the cell indicates the total intensity of the cell minus the intensity of the region of the cytoophidium. Scale bars = 20 μm.

**Figure 9:**
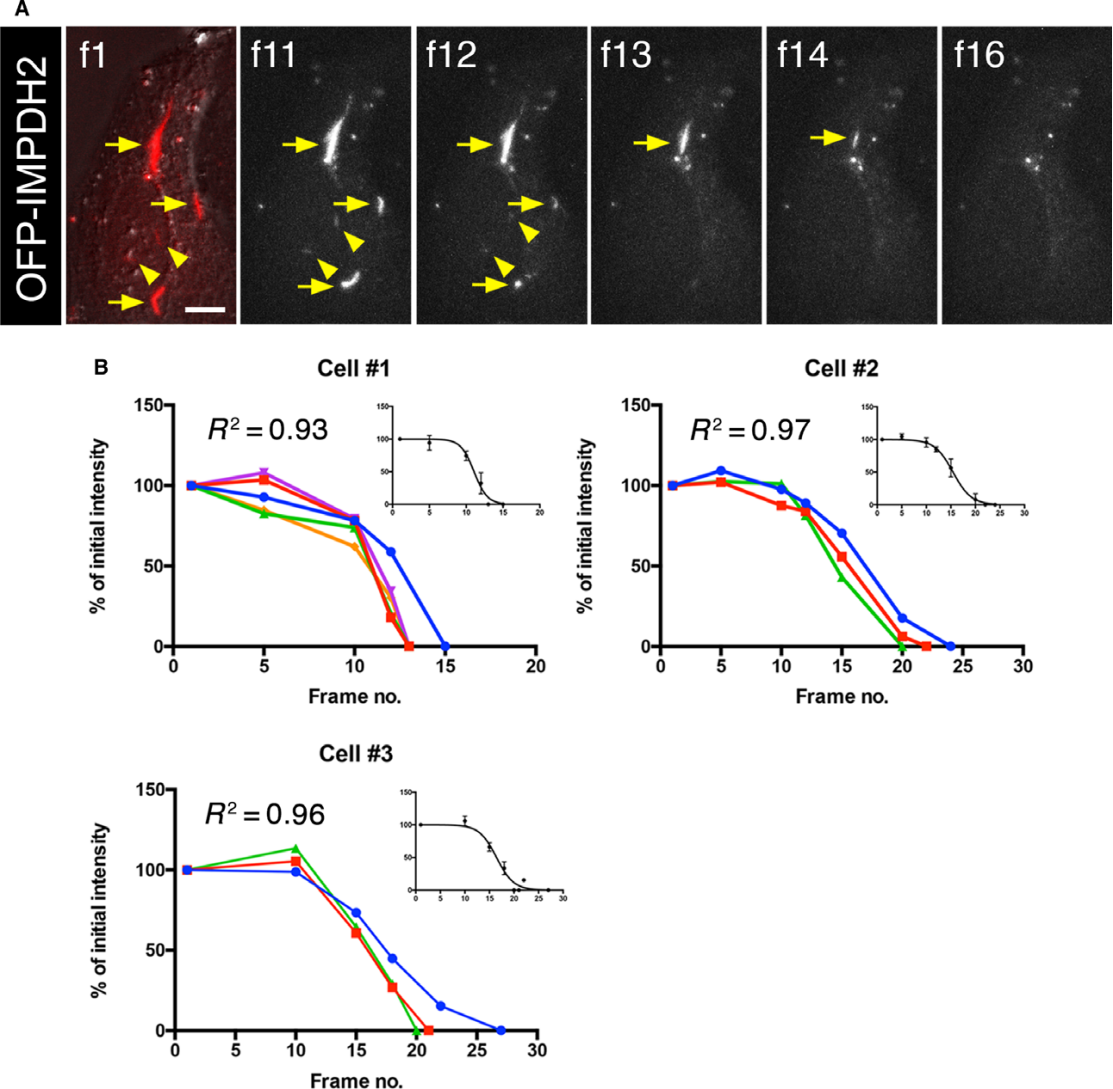
Synchronous disassembly of multiple IMPDH in mixed cytoophidia in single cell. (A) Representative frames of video show the disassembly of DON‐induced cytoplasmic (arrows) and nuclear (arrowheads) IMPDH cytoophidia by guanosine treatment. Scale bar = 10 μm. (B) The quantification of fluorescence intensity of OFP‐IMPDH2‐labelled cytoophidia in three individual cells with multiple cytoophidia (including cytoplasmic and nuclear cytoophidia).

The processes of cytoophidium assembly have been determined previously 5, 24, 25. However, it is still unclear how the cytoophidium disassembles in mammalian cells. Drug‐induced IMPDH cytoophidium formation is generally reversible by additional guanosine or GTP in the culture medium 1, 2. Thus, we sought to capture the image of disassembly of the IMPDH cytoophidium under such conditions. Firstly, we induced cytoophidium formation in OFP‐IMPDH2/CTPS1‐GFP‐expressing model cells with DON. After an overnight treatment, massive mixed cytoophidia were present in most of cells (Fig. 6A f1). We then added guanosine to the medium and started recording. Approximately 20 min later, IMPDH cytoophidia started to disassociate and the OFP signal resident in filament structures gradually faded out (Fig. 6A f9–f26). Within one hour, all IMPDH cytoophidia had disappeared without a noticeable reduction of GFP signal within the original cytoophidia (Fig. 6B). A similar pattern was also observed in cells treated with DAU (Figs 5 and 7). Interestingly, although the length or intensity of the CTPS cytoophidium was not affected by the loss of its IMPDH partner, the morphology was changed in some cases. For instance, as shown in Fig. 7 and Video S6, a ring‐shaped cytoophidium turned into linear soon after loss of its IMPDH counterpart. Moreover, in order to understand whether IMPDH within the cytoophidium was just released from the aggregates but not degraded while the filament disassembles, we quantified the fluorescence intensity of MPA‐treated OFP‐IMPDH2‐expressing HeLa cells (excluding the intensity of visible filaments), in time‐lap pictures of IMPDH cytoophidium disassembly by guanosine supplementation. Six samples in the images were analysed. Five cells show gradually increased fluorescence intensity at each time point of IMPDH cytoophidium disassembly, and the other one (Cell #4), which has the lower fluorescence intensity at region of the cytoophidium, show only small changes in the intensity of the cell by the time. This result indicates that IMPDH filaments turn back to diffused proteins after the guanosine supplementation (Fig. 8). We also analysed the fluorescence intensity of each IMPDH cytoophidium in DON‐treated cells at different time points upon guanosine supplementation. The result shows that the disassembly of all IMPDH cytoophidia in the same cell took place simultaneously, no matter the differences in their sizes, morphology and localisation, indicating a precise control of filamentation by the increase in intracellular GTP level (Fig. 9).

### Turnover of subunits within the cytoophidium

**Figure 10:**
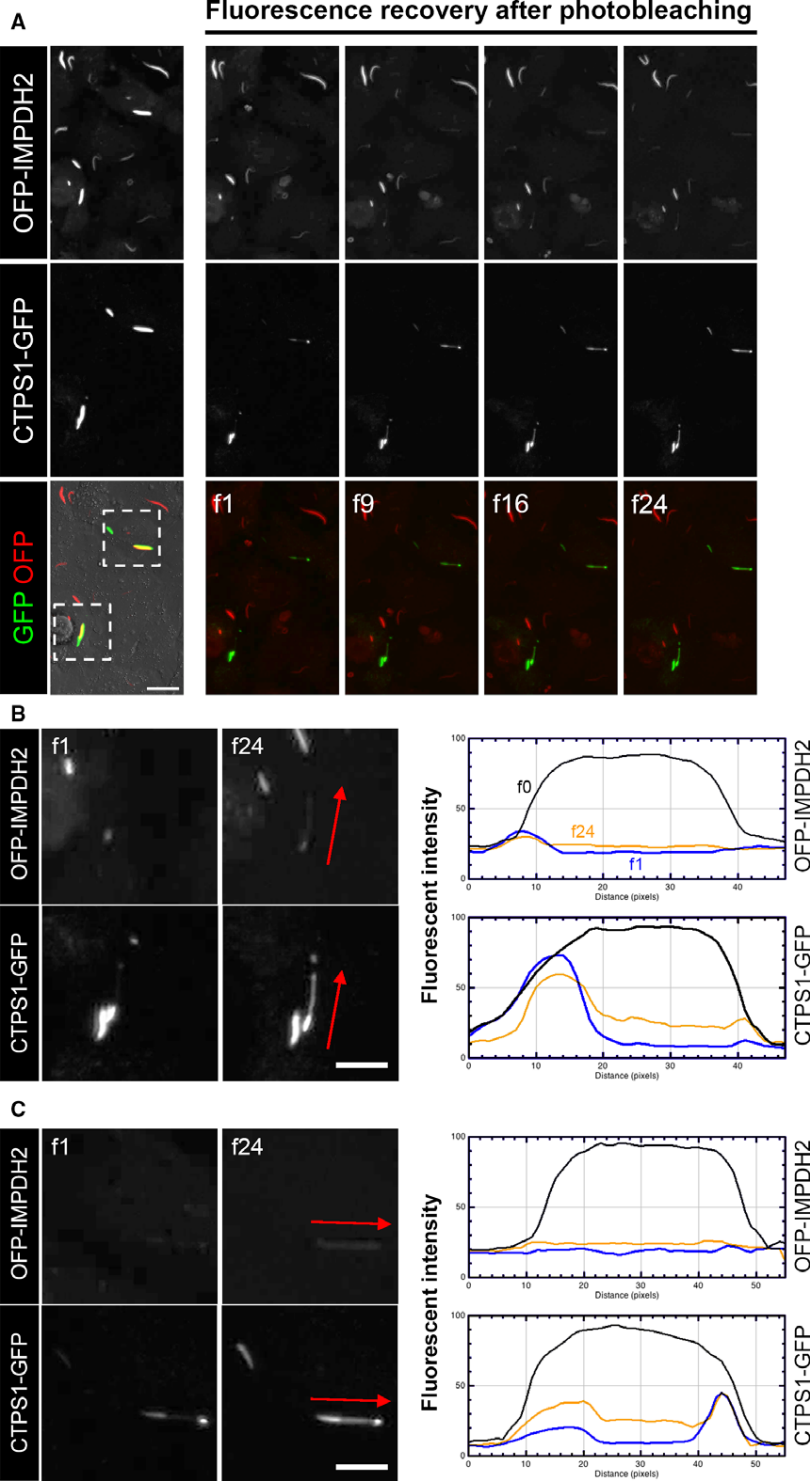
FRAP of cytoophidia. (A) Representative frames of a video with frame numbers as indicated. Fluorescence of DON‐induced mixed cytoophidia was bleached with intensive laser stimulation and the video recording was carried out upon photobleaching with a 2‐min interval between each frame. Scale bar = 20 μm. (B,C) Magnified image of areas indicated in (A) and the quantification of fluorescence intensity for OFP and GFP in an individual cytoophidium in corresponding frames. Arrows indicate the direction for fluorescence intensity analysis. Scale bars = 10 μm.

In order to investigate whether proteins building the cytoophidium have an active turnover, we carried out a fluorescence recovery after photobleaching (FRAP) approach on OFP‐IMPDH2/CTPS1‐GFP‐expressing cells. We treated model cells with DON for 1 day to induce mixed cytoophidia. Subsequently, we bleached cytoophidia with single or dual fluorescent signals in part of or the entire structure, and started video recording upon photobleaching. In the mixed cytoophidia, both fluorescent signals gradually recovered over time (Fig. 10). With the quantification of fluorescent intensity of an individual cytoophidium at different time points, we show that recovery of the CTPS1‐GFP signal in the cytoophidium is faster than that of OFP‐IMPDH2, maybe because OFP‐IMPDH2 was sorted for medium level of fluorescence intensity stable expression, while CTPS1‐GFP is a transient expression. Additionally, fluorescence was recovered evenly and no apparent change in length of unbleached parts at the two ends was observed (Fig. 10B,C). These findings suggest that the cytoophidium may continuously renew its subunits in the presence of stimulation and, moreover, that there is no polarity in the cytoophidium structure for protein turnover, unlike for example cytoskeleton structures, which have positive and negative ends.

### The super‐resolution ultrastructure of IMPDH and CTPS mixed cytoophidium

**Figure 11:**
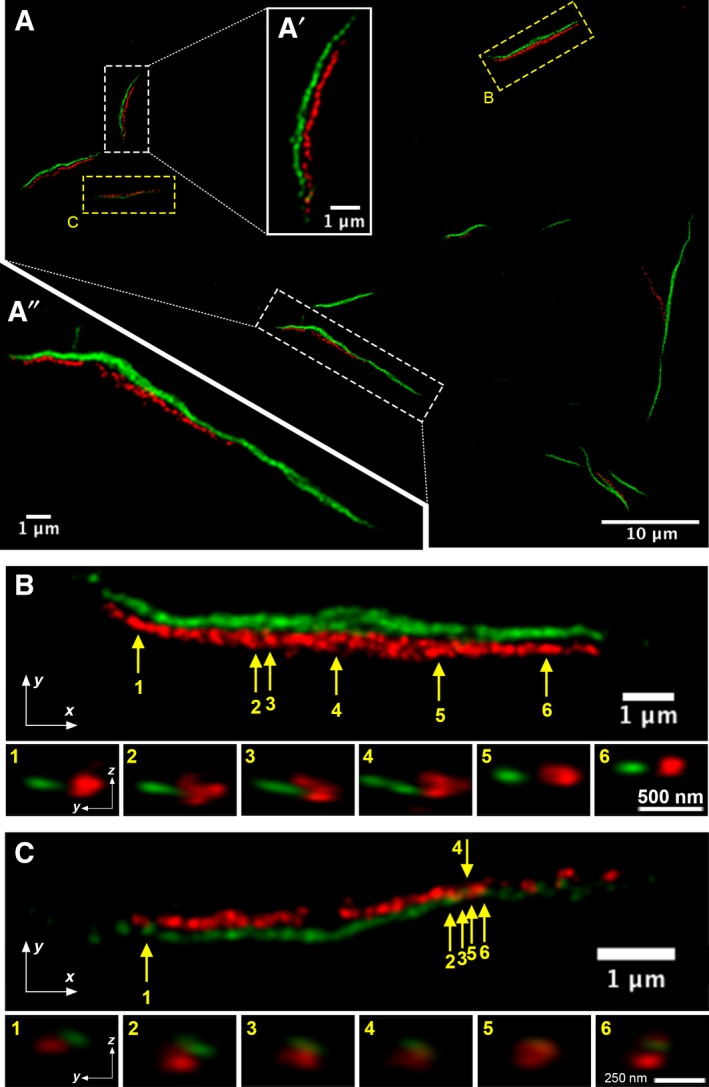
Ultrastructure of IMPDH/CTPS mixed cytoophidium. (A) STED microscopic image of several IMPDH/CTPS mixed cytoophidia. IMPDH is shown in red and CTPS is shown in green. (A′) and (A″) are magnified images of selected region in (A). (B) and (C) show magnified images of two cytoophidia selected in (A). Arrows and numbers indicate the points for resection in side view and the images of corresponding sections are shown below.

We have demonstrated in our previous study that mixed cytoophidia account for about 30% of all cytoophidia found in HeLa cells treated with DON 20. However, data from the current study suggests that CTPS and IMPDH filaments within the same cytoophidium could be independent structures. It is intriguing to know how filaments of two enzymes coordinate in a macrostructure. According to electron microscopic images of the ultrastructure of the IMPDH cytoophidium, the single filament consists of a bundle of a massive number of protein polymers, or primary fibres 21. This remarkable finding gives us the concept about the basis of the cytoophidium. However, it is not possible to determine the association between IMPDH and CTPS filaments from such images. This prompted us to reveal the ultrastructure of mixed cytoophidia with a stimulated emission depletion (STED) super‐resolution microscope. To achieve this, OFP‐IMPDH2 and CTPS1‐GFP coexpressing HeLa cells were treated with DON for 1 day and subsequently fixed and labelled with anti‐IMPDH1 and anti‐CTPS1 antibodies to further enhance the fluorescence signal. Interestingly, in all mixed cytoophidia examined, CTPS and IMPDH filaments were actually separate but aligned or intertwined with one another (Fig. 11A). The width of filaments ranged from ~ 100 nm to ~ 300 nm, which is consistent with observations under the electron microscope 21. Similar to the macrocytoophidium in the *Drosophila* egg chamber 12, loosening of a CTPS filament could be seen occasionally (Fig. 11A″,B). In some cases, a gap between two aligned filaments could be clearly observed, suggesting CTPS and IMPDH filaments may not directly interact with one another (Fig. 11A′,B,C). Our findings indicate that IMPDH and CTPS can form primary fibres (polymer) and larger filaments (the bundle of primary fibres) independently, and associate with another individual filament in a higher order structure of the cytoophidium.

## Discussion

Human IMPDH octamer and CTPS tetramer are known as their active states, but recent studies have demonstrated that human IMPDH1 octamers and CTPS1 tetramers alone are able to build up polymer structures *in vitro* under certain circumstances 8, 10, 28, 29. For instance, presence of MgATP and an adRP10‐related mutation, D226N, promotes human IMPDH1 polymerisation and further aggregation *in vitro*, while *in vitro* polymerisation of human CTPS1 could be promoted by its substrate UTP 8, 10. Such polymers have been considered to be the primary structure of the cytoophidium according to electron microscopic observations 13, 21.

Several compounds, mostly inhibitors for nucleotide synthesis, have been shown to induce cytoophidium formation and been widely used for the investigation of cytoophidium features. DON is known as the most effective inducer for both CTPS and IMPDH cytoophidium as it can induce massive filamentation of two enzymes within an hour 2, 3, 5. It blocks nucleotide and protein synthesis by inhibiting a variety of enzymes, including FGAR amidotransferase, asparagine synthetase carbamoyl phosphate synthetase and CTPS 30, 31, 32. In contrast, DAU and MPA affect specifically to their targets CTPS and IMPDH, respectively. DAU could be converted into deaza‐UTP, an analogue of UTP, and subsequently, perform competitive inhibition on CTPS 33. This action suppresses *de novo* pyrimidine synthesis and thereby increases purine nucleotide production 7. This also indirectly triggers IMPDH filamentation within a short period until too much GTP is accumulated in the cell 7. MPA is an uncompetitive and reversible inhibitor of IMPDH. It interferes with substrate turnover of both IMPDH isoforms by direct binding resulting in a significant decrease of intracellular GTP level 34. Intensive investigation for the regulation of IMPDH and CTPS filaments has been done *in vitro* and in cell‐free systems, the details of action have been discussed in previous reports [4, 7, 8, 9, 10, 19, 28].

The cytoophidium is also termed ‘rods and rings’ in some studies because some cytoophidia display a linear appearance, while some may seem as a circle or a ring 2. In most cell types, the linear cytoophidium is the major type. However, more than 80% of IMPDH cytoophidia were observed in ring‐shaped in mouse embryonic stem cells 2. It is intriguing that whether the rod‐like and ring‐like structures function and behave differently. With the help of live‐cell imaging, we have captured movies of the formation and movement of IMPDH cytoophidia. Our observation suggests that the cytoophidium with different morphology may be substantially identical as fusion of two or more cytoophidia occurs between different types of cytoophidia.

**Figure 12:**
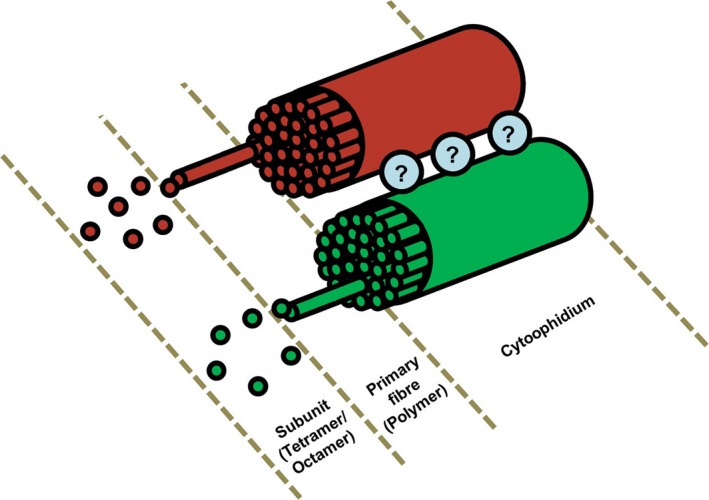
Model of the organisation of the cytoophidium structure. An illustration of the ultrastructure of the IMPDH/CTPS mixed cytoophidium. The ‘?’ factor represents the unknown component in the cytoophidium in charge of connecting the IMPDH and CTPS components.

To date, CTPS cytoophidium has been identified in several organisms including prokaryotes and eukaryotes 3, 11, 12, 13. The IMPDH cytoophidium, however, has only been observed in mammals 1, 2. In several mammalian cell lines, such as HEK 293T cells, HEp‐2 cells, COS‐7 cells and HeLa cells, the CTPS and IMPDH cytoophidium could exist individually in the same cell, while mixed cytoophidia could also be seen frequently 2, 7, 20. Besides, filamentation of CTPS and IMPDH is regulated independently as some drugs, such as MPA and ribavirin, could only trigger the filamentation of IMPDH, while DON and DAU could induce both cytoophidia 7, 20. These findings indicate the CTPS filament is not necessary for the formation of IMPDH cytoophidium, and vice versa. In the current study, our results suggest an interaction may exist between CTPS and IMPDH cytoophidium, as colocalised CTPS and IMPDH cytoophidia often move synchronously, and the fission and fusion between both filaments were also observed. Furthermore, the disassembly of the IMPDH cytoophidium may not affect the appearance of its CTPS cytoophidium counterpart. By applying STED microscopy, we confirmed the mixed cytoophidium is actually formed by two or more individual IMPDH and CTPS cytoophidium lying closely with a tiny gap in between. Accordingly, we speculate filaments of both enzymes may share a mechanism or bind with a common interacting factor (the ‘glue’ factor shown in Fig. 12) to support the macrostructure or for the guidance of its distinctive movement. Although the IMPDH cytoophidium was not enriched in actin, tubulin or vimentin and not associated with centrosomes or other known cytoplasmic structures in mammalian cells, CTPS filaments of *Caulobacter crescentus* are associated with the intermediate filament, crescentin, thereby regulate the curvature of *C. crescentus* cells 2, 13. In addition, despite the IMPDH cytoophidium is not suggested as a membrane‐bound structure under the observation with electron microscopy 21, we still could not exclude the possibility that cytoophidium movement might be linked with membrane dynamics. It is reasonable to speculate certain structural proteins or even intracellular membrane may associate with the cytoophidium in mammalian cells as well.

As shown in our previous studies, assembly of CTPS and IMPDH cytoophidium is a natural phenomenon that takes place in some normal and cancerous tissues of mouse and human without additional drug inductions 6, 7. As the formation of polymers of CTPS and IMPDH has been considered a mechanism for modulating protein properties such as catalytic activity, the knowledge of the regulation and function of given subcellular structures may provide new insights on cell metabolism 10, 17, 35, 36. Recently, the structures of human CTPS1 and IMPDH2 polymers and the regulation of their assembly *in vitro* have been revealed with electron microscopy 10, 19. However, observations on purified proteins *in vitro* could not fully explain how such polymers further aggregate into the macrostructure as seen in cells. Herein, our results explore the dynamics of the CTPS and IMPDH mixed cytoophidium in live cells, its assembly and disassembly, which is not available through the immunostain‐based analysis. Using FRAP experiments, we shown that the cytoophidium may continuously renew its subunits in the presence of stimulation and there is no polarity in the cytoophidium structure for protein turnover. We also reveal the ultrastructure of the mixed cytoophidium and propose the cytoophidium is not a simple aggregate of protein polymers. Our findings provide valuable information for future studies on discovery of other components in the cytoophidium and the underlying mechanisms, which may subsequently shed the light on the regulation and physiological purpose of this subcellular macrostructure.

## Materials and methods

### Cell culture

Human HeLa cells (Culture Collections, Public Health England, 93021013) were cultured in Dulbecco's modified Eagle's medium with high glucose, glutamine (Thermo Fisher Scientific, Paisley, UK), 1% Gibco^®^ Antibiotic‐Antimycotic (Thermo Fisher Scientific) and 10% foetal bovine serum (Thermo Fisher Scientific). Cells were kept in a 37 °C humid incubator with 5% CO_2_. DAU (Sigma‐Aldrich, Dorset, UK), MPA (Sigma‐Aldrich) and guanosine (Sigma‐Aldrich) were dissolved in DMSO (Sigma‐Aldrich) and DON was dissolved in water. Given compounds were added to cultured medium as described in individual experiments.

### Constructs and transfection

Human IMPDH2 and mouse CTPS1 coding sequences were cloned into pCMV3‐N‐OFPSpark (Sino Biological, Beijing, China; HG14878‐ANR) and pLVX‐EF1alpha‐AcGFP‐N1 (Clontech, Mountain View, CA, USA; 631983) vectors, respectively. P2A sequence was inserted between OFPSpark and IMPDH2 to generate pCMV3‐OFPSpark‐P2A‐IMPDH2 construct with Gibson Assembly System (NEB). Cell transfection was done with lipofectamine 3000 reagent (Thermo Fisher Scientific) or Effectene Transfection reagent (Qiagen, Hilden, Germany) according to instructions provided by the manufacturers.

HeLa cells transfected with OFP‐IMPDH2 construct and selected with 2 μg·mL^−1^ of Hygromycin B for 2 weeks were sorted according to OFPSpark fluorescence intensity using a MoFlo Astrios cytometer (Beckman Coulter, Brea, CA, USA).

### Immunoblotting

Total cell extract was quantitated for the amount of protein using a BCA Protein Assay Kit (Thermo Fisher Scientific). About 10 μg of protein was loaded in each well of 15 per well NuPAGE Bis‐Tris gels, run with XCell SureLock Mini‐Cell Electrophoresis System and transfer to nitrocellulose membrane with XCell II Blot Module (Thermo Fisher Scientific). For immunolabelling, primary and secondary antibodies were incubated overnight diluted in TBS + 5% milk. Antibody labelling was revealed with SuperSignal West Pico Chemiluminescent Substrate (Thermo Fisher Scientific) and visualised in a G:BOX Chemi XT4 machine (Syngene, Cambridge, UK). Antibodies used: rabbit polyclonal anti‐IMPDH2 (1 : 1000, ProteinTech, Chicago, IL, USA; 12948‐1‐AP); HRP‐conjugated mouse monoclonal anti‐ACTB (1 : 3000, ProteinTech, HRP‐60008). HRP‐conjugated donkey anti‐rabbit IgG (1 : 1000, Jackson ImmunoResearch, Cambridgeshire, UK; 711‐035‐152).

### Immunofluorescence

Cells were fixed with 4% paraformaldehyde in PBS for 10 min. Fixed samples were incubated in PBS staining buffer containing 2.5% horse serum, 0.25% Triton X‐100 (Sigma‐Aldrich) and primary antibody at room temperature for more than two hours. After washing with PBS, samples were incubated in staining buffer with secondary antibody at room temperature for 2 h. Antibodies used in this study are as follows: rabbit polyclonal anti‐CTPS1 (1 : 500, ProteinTech, 15914‐1‐AP), rabbit polyclonal anti‐IMPDH2 (1 : 500, ProteinTech, 12948‐1‐AP), mouse monoclonal anti‐IMPDH1 (1 : 500, Abcam, Cambridge, UK; ab55294), Alexa Fluor 488‐conjugated donkey anti‐rabbit IgG (1 : 500, Invitrogen, Paisley, UK; A‐21206) and Cy3‐conjugated donkey anti‐mouse IgG (1 : 500, Jackson ImmunoResearch, 715‐485‐151).

### Microscopy

Images were acquired under 63× objectives on a confocal microscope (Leica TCS SP5 II confocal microscope, Milton Keynes, UK). Super‐resolution images were acquired with a STED confocal microscope (Leica SP8 Gated STED).

### Live imaging

HeLa cells transfected with OFP‐IMPDH2 and CTPS1‐GFP constructs were cultured on glass bottom culture dishes (MatTek Corporation, Bratislava, Slovakia; P35G‐1.5‐10‐C) with medium containing 10 mm HEPES (Sigma‐Aldrich, 83264), and maintained at 37 °C when live imaging was performed.

### Image analysis

Fluorescence intensity of images was analysed with the software imagej (NIH, Bethesda, MD, USA). For quantification of fluorescence intensity shown in Fig. 8, the margin of each cell and IMPDH cytoophidium was selected manually. The correlation coefficient of the intensity of frames was analysed with Graph Prism 6.

## Author contributions

CCC, GDK, LYS and JLL conceived this project; CCC and GDK designed and performed experiments. CCC, GDK, LYS and JLL analysed the data; CCC and GDK wrote the manuscript with input from LYS and JLL.

## Conflict of interests

The authors declare no conflict of interest.

## Supporting information


**Video S1.** Transfiguration of an IMPDH cytoophidium.

**Video S2.** Maturation of linear and ring‐shaped cytoophidia.

**Video S3.** Fission and fusion of ring‐shaped cytoophidia.

**Video S4.** Formation and separation of IMPDH and CTPS filaments.

**Video S5.** Separation of a CTPS filament from an IMPDH/CTPS mixed cytoophidium.

**Video S6.** Disassembly of IMPDH filaments in a mixed cytoophidium.
